# Soft Tissue Dimensions Following Tooth Extraction in the Posterior Maxilla: A Randomized Clinical Trial Comparing Alveolar Ridge Preservation to Spontaneous Healing

**DOI:** 10.3390/jcm9082583

**Published:** 2020-08-10

**Authors:** Young Woo Song, Sung-Wook Yoon, Jae-Kook Cha, Ui-Won Jung, Ronald E. Jung, Daniel S. Thoma

**Affiliations:** 1 Department of Periodontology, Research Institute for Periodontal Regeneration, Yonsei University College of Dentistry, Seoul 03722, Korea; tigger09@yuhs.ac (Y.W.S.); sungwook08@yuhs.ac (S.-W.Y.); chajaekook@yuhs.ac (J.-K.C.); daniel.thoma@zzm.uzh.ch (D.S.T.); 2 Clinic of Reconstructive Dentistry, Center of Dental Medicine, University of Zurich, 8032 Zurich, Switzerland; ronald.jung@zzm.uzh.ch

**Keywords:** alveolar ridge preservation, gingival thickness, cone-beam computed tomography, stereolithography, superimposition

## Abstract

Background: To assess the soft tissue dimension following tooth extraction and alveolar ridge preservation in the posterior maxilla compared to spontaneous healing. Methods: Thirty-five patients randomly assigned to alveolar ridge preservation (ARP) and spontaneous healing (SH) after maxillary molar extraction. The crestal, buccal, and palatal gingival thickness at 6 months was measured around virtually placed implant fixtures using superimposed cone-beam computed tomography and intraoral scan taken at 6 months. Buccal mucogingival junction (MGJ) level change over 6 months was estimated using intraoral scans obtained at suture-removal and 6 months. Results: The crestal gingiva was significantly thinner in group ARP (−1.16 mm) compared to group SH (*p* < 0.05). The buccal and palatal gingiva was significantly thinner at the implant shoulder (IS) level in group ARP (buccal: −0.75 mm; palatal: −0.85 mm) compared to group SH (*p* < 0.05). The thickness at 2 mm below the IS of both sides and the buccal MGJ level change were similar in both groups (*p* > 0.05). Conclusions: ARP in the posterior maxilla resulted in a thinner soft tissue on top of and at the prospective level of the implant shoulder at 6 months. The buccal MGJ level changed minimal for 6 months in both groups.

## 1. Introduction

Alveolar ridge preservation (ARP) following tooth extraction is a well-documented clinical procedure with a number of reported benefits including: limiting morphologic changes of the ridge contour, preserving the integrity of hard and soft tissues [1], optimizing the ridge contour in extraction sites with a loss of the buccal bone plate [2,3], preventing shrinkage of the keratinized tissue [4], and resisting maxillary sinus floor pneumatization in case of extractions in the posterior maxilla [5,6]. Moreover, ARP can compensate for alveolar bone resorption and changes of the soft tissue contour that spontaneously occur following tooth extraction [7,8,9].

Based on the previous studies, the soft tissue thickness can be significantly influenced by the underlying hard tissue dimension following single tooth extraction in the anterior area [10,11]. These results obtained by three-dimensional analyses suggested that the soft tissue thickness increased significantly in case of alveolar bone resorption and only minimally changed in case of a well-preserved hard tissue dimension.

An increased soft tissue thickness is of benefit in terms of post-surgical healing, since it has more vascularization and a higher volume of extracellular matrix within the connective tissue layer [12]. The thickness of the soft tissue covering the extracted site has been considered important, since it could influence the thickness of the mucosa following implant placement, which eventually influences the health of peri-implant tissue. Biologic width or supracrestal tissue height, measured by the total length of the sulcus depth, junctional epithelium, and connective tissue contact [13], is physiologically formed and stable structure that can provide biological barrier against microbial invasion [14,15]. Although varying distance of biologic width around dental implants have been suggested in previous studies, ranging from 3 to 4 mm [16], it is known that peri-implant tissues undergo physiological alterations such as marginal bone resorption to achieve adequate thickness in cases of thin supracrestal peri-implant mucosa [14].

A number of previous studies showed a reliable performance of ARP in terms of preserving the crestal height [2,6,17,18,19]. A recently published randomized controlled clinical trial also demonstrated the prevention of maxillary sinus floor augmentation, which consequently resulted in optimization of vertical dimension and reducing the necessity of excessive sinus graft surgery [6]. The change in the soft tissue following ARP has been well described in the esthetic zone previously [11]; there are, however, a limited number of studies reporting on the dimension of soft tissue investigated after ARP in the posterior maxilla.

Therefore, the purpose of present study is to assess the soft tissue dimension following tooth extraction and alveolar ridge preservation in the posterior maxilla compared to spontaneous healing based on three-dimensional intraoral scan data and cone-beam computed tomography (CBCT).

## 2. Experimental Section

### 2.1. Study Design and Population

**Figure 1:**
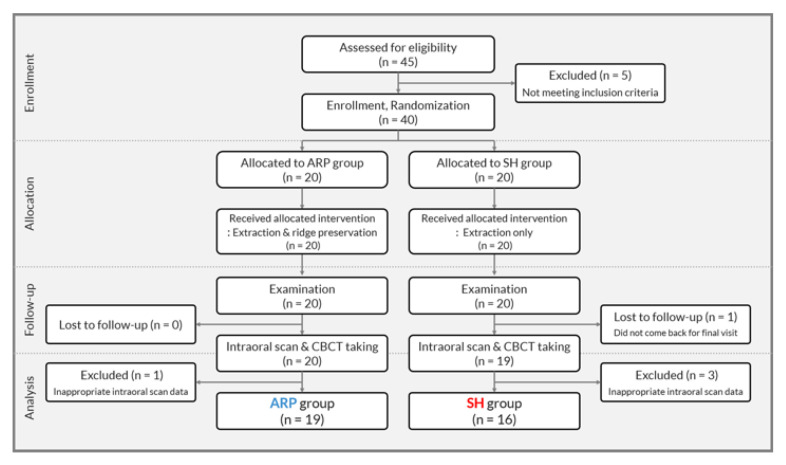
CONSORT flowchart of the study.

The present study prospectively investigated the dimensional changes of soft tissues of subjects having been enrolled in a randomized controlled clinical trial [6]. The ethical approval of the trial was obtained from the Institutional Review Board of Yonsei University Dental Hospital (No. 2-2016-0033), and the study was registered with the Clinical Research Information Service of National Research Institute of Health in Republic of Korea (KCT0003252). The CONSORT flowchart is presented in Figure 1 [20].

### 2.2. Inclusion and Exclusion Criteria

The inclusion and exclusion criteria of the randomized controlled clinical trial in detail are described in the previously published article [6]; in brief, patients who needed the extraction of one or more maxillary molars. All of the included subjects were ≥18 years of age without any systemic or local conditions contraindication to surgical treatment and had more than two adjacent teeth maintained to allow superimposing digital scans. Exclusion criteria were patients with a systemic disease or bone metabolic disorder, a history of malignancy, radiotherapy, or chemotherapy in the past 5 years and a heavy smoking habit (>20 cigarettes daily) as well as pregnant or lactating patients.

### 2.3. Group Allocation and Sample Size

All enrolled subjects were randomly allocated to one of the following groups, either ARP group or spontaneous healing (SH) group for the treatment [6].

- ARP group: ARP performed immediately after extraction of upper posterior teeth;
- SH group: No additional grafting, but spontaneous healing following extraction.

The total sample size of 40 (20 subjects for each group) was determined based on the previously reported hard tissue dimensional change following ARP [21] with a significance level of 0.05 and a power of 80% [6]. Among 39 subjects (20 ARP group patients and 19 SH group patients) who finished the clinical trial, 35 patients (19 ARP group patients and 16 SH group patients) were eligible for the present investigation having intraoral scan data without artifacts obtained at 6 months post-surgery. Four patients (1 ARP group patient and 3 SH group patients) were excluded, since their intraoral scan data presented image voids and distortions that interfered with the superimposition. The acquired sample size of 35 (19 patients in ARP group and 16 patients in SH group) was confirmed sufficient in post hoc based on the previously reported result on gingival thickness post tooth extraction [11], since 16 subjects per group were estimated as a minimal number of participants with the significance level of 5% and the power of 95%.

### 2.4. Randomization and Blinding

Immediately after tooth extraction, all enrolled patients were randomly allocated to either the ARP or SH group using a sealed envelope prepared by a web-based computer software (sealedenvelope.com).

### 2.5. Treatment Procedures and Post-Surgical Evaluations

**Figure 2:**
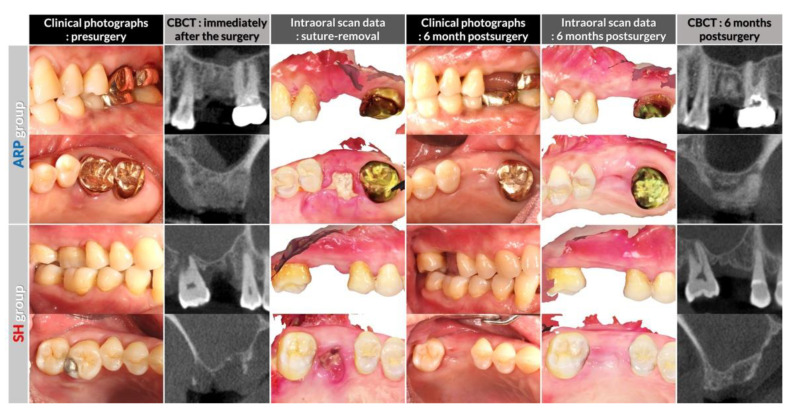
Representative clinical photographs, intraoral scan and conebeam-computed tomography (CBCT) images.

After scaling and root planning at screening, maxillary molars were gently extracted by a flapless procedure at the second visit. While SH group patients received the extraction only, a collagenated deproteinized bovine bone mineral (BioOss Collagen®, Geistlich, Wolhusen, Switzerland) was grafted in the extraction socket, and a resorbable native, non-crosslinked collagen membrane (BioGide®, Geistlich) was applied to cover the bone graft material in group ARP. There was no attempt to achieve primary wound closure in both groups. Resorbable sutures, however, were used to adapt the wound margins in both groups (Monosyn 4-0® Glyconate Monofilament, B. Braun Tuttlingen, Melsungen, Germany). Antibiotics (amoxicillin 500 mg) and analgesics (ibuprofen 200 mg) were given to the patients, three times daily for 5 days. Sutures were removed at 7–12 days post-surgery, and follow-up examinations were performed at 1, 3, and 6 months after the extraction. A baseline CBCT was obtained immediately after the surgery, and baseline intraoral scan was achieved at suture-removal. At 6 months, the final visit of the trial, a CBCT and an optical impression were taken (Figure 2).

### 2.6. Outcome Variables

- Primary outcomes: Vertical thickness of crestal gingiva measured at 6 months post-surgery.
- Secondary outcomes: Tangential thickness of the buccal and palatal gingiva measured at 6 months post-surgery; change of the level of the buccal mucogingival junction (MGJ) from suture-removal to 6 months.

### 2.7. Data Collection, Virtual Implant Planning, and Outcome Measurements

All measurements were performed by a single, experienced investigator (S.-W.Y.), who was blinded to the group allocation.

#### 2.7.1. Vertical Thickness of the Crestal Gingiva at 6 Months

**Figure 3:**
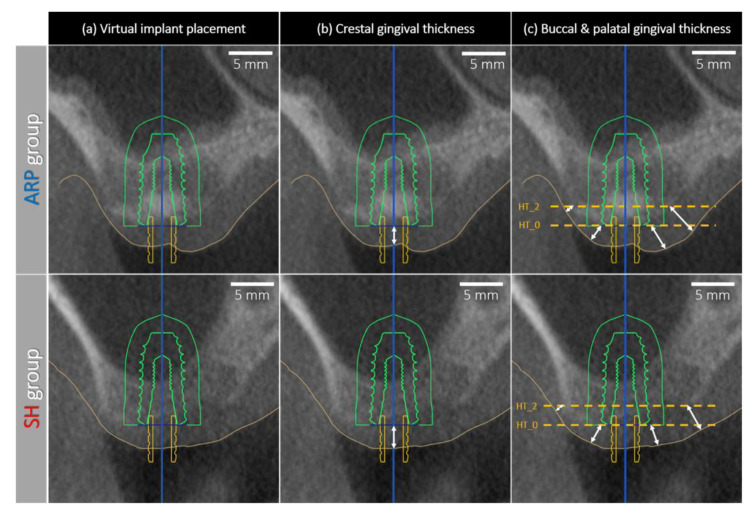
Measurement of gingival thicknesses at 6 months post-surgery. (**a**) Virtual placement of the implant fixture. (**b**) Vertical thickness of crestal gingiva was measured from the bone crest to gingival margin (white arrow) along the midline of the virtually placed implant fixture (blue line). (**c**) Reference lines (yellow-dotted lines) for the measurement of tangential thicknesses at two different level, implant shoulder level (HT_0) and 2 mm below the implant shoulder (HT_2), were drawn perpendicular to the midline of the virtually placed implant fixture. Buccal and palatal gingival thicknesses were measured diagonally (white arrows) from the bone crest to the tangent of gingival margin.

The vertical thickness of the crestal gingiva was measured using a digital implant planning program (Implant studio^TM^, 3 shape, Copenhagen, Denmark). The CBCT data and stereolithograph (STL) files obtained by an intra-oral scanner (Trios, 3 shape) taken at 6 months after the surgery were superimposed in the software program (Implant studio^TM^, 3 shape) using three or more common landmarks of adjacent teeth [22]. An implant fixture of 5 mm in diameter and 10 mm in length was virtually placed in a prosthetically appropriate position, positioning the platform at the bone crest level (Figure 3a). In case, the level of the buccal and palatal bone crest differed, the fixture platform was virtually placed at the more coronal level of the buccal or palatal side, assuming that a bone augmentation procedure would be performed for the peri-implant dehiscence defect. The height of the soft tissue was measured vertically from the bone crest to the mucosal margin of the extraction socket along the midline of the virtually placed implant (Figure 3b).

#### 2.7.2. Tangential Thickness of the Buccal and Palatal Gingiva at 6 Months

The STL and CBCT data were superimposed (Implant studio^TM^, 3 shape) in order to assess the thickness of the buccal and palatal gingiva. For that purpose, the center of the virtually placed implant fixture served as a vertical reference. The extended line perpendicular to the vertical reference line and coinciding the shoulder of the implant fixture served as a horizontal reference. On both, the buccal and palatal side, the thicknesses were measured by drawing a tangent line between the bone and gingival margin at two different levels as follows (Figure 3c):

- HT_0: The level of the implant shoulder;
- HT_2: The level of 2 mm below the implant shoulder.

#### 2.7.3. Change of the Level of the MGJ on the Buccal Side from Suture Removal to 6 Months

**Figure 4:**
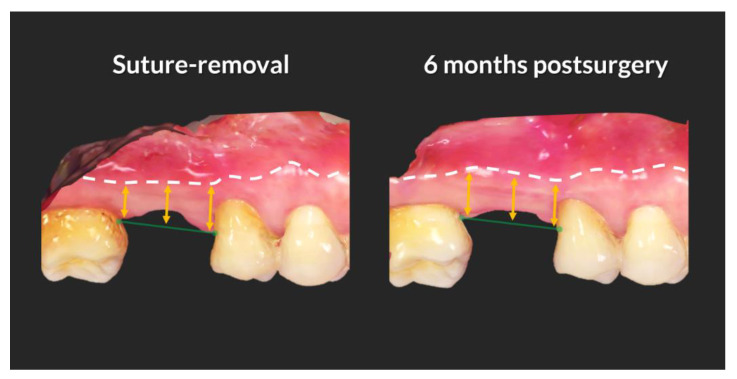
Measurement of buccal mucogingival junction (MGJ) level change over 6 months. At both time point (suture-removal and 6 months post-surgery), vertical height (yellow arrow) from the extended line of proximal cemento-enamel junctions of adjacent teeth (green line with round arrowheads) to MGJ (white-dotted line) was measured in the mesial-most, center-most, and distal-most point, and the mean of the measurements was calculated at each time point. The mean height of suture-removal was subtracted from that of 6 months post-surgery to derive vertical level change of MGJ of each case over 6 months.

The position of the buccal MGJ was measured from the extended line of the proximal cemento-enamel junctions (CEJs) of the adjacent teeth to the MGJ, using intra-oral scan data taken at suture-removal and at 6 months. Buccal aspects of the extraction sites were screen-captured with a 2-mm reference line on a STL file analyzing software (OrthoAnalyzer^TM^, 3 shape). Mesial, center and distal levels of the screen-captured images were measured separately at suture-removal and at 6 months using a software (Image J; National Institute of Health, Bethesda, MD, USA), and the mean value at each time point was estimated for each subject. Thereafter, the changes between the two time-points were calculated by subtracting the mean value of suture-removal from that of 6 months post-surgery. Positive value meant that MGJ moved apically, whereas the negative value indicated coronal shifting of MGJ (Figure 4).

### 2.8. Statistical Analysis

The statistical analysis was performed using a computer software (SPSS version 23, IBM, Armonk, NY, USA). The normality of the data was confirmed by Shapiro-Wilk test (*p* > 0.05), therefore test group and control group were compared by independent t test. The results are presented as mean values with standard deviations, and the level of significance was set *p* < 0.05.

## 3. Results

### 3.1. Demographic Information of Participants and Clinical Findings

The mean age of 35 patients was 55.3 ± 8.33 years (ARP; n = 19) and 50.8 ± 12.6 years (SH; *n* = 16), respectively. Every subject who was included for the evaluation in the present study received a single molar extraction, and therefore 35 molars were assessed. Fourteen first molars and 5 s molars in group ARP and 7 first molars and 9 s molars in group SH were included in the analyses. Five cases in group SH demonstrated peri-implant dehiscence defects (1 buccal defect of 2 mm in height; 4 palatal defects of 1 mm in height), whereas three cases demonstrated a palatal dehiscence defect of 1 mm in height in group ARP (Table 1).

Among the 35 subjects, none showed complications following tooth extraction, except for one patient in group ARP demonstrating a partial exposure of the bone graft material and the buccal bone plate at suture-removal. Two weeks later, the wound was closed without further intervention.

### 3.2. Outcome Measurements

#### 3.2.1. Vertical Thickness of Crestal Gingiva at 6 Months

**Figure 5:**
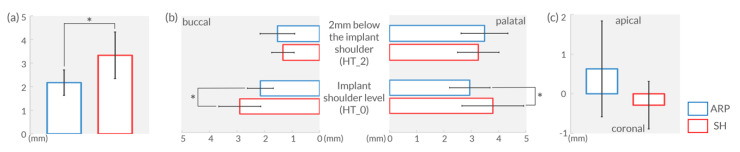
Results of the measurements. (**a**) Vertical thickness of crestal gingiva at 6 months post-surgery. (**b**) Tangential thicknesses of buccal and palatal gingiva at 6 months post-surgery. (**c**) Buccal mucogingival junction level change over 6 months. Asterisks. * represent statistical significance observed between ARP and SH groups (*p* < 0.05).

In the center of the site (virtual implant position), the thickness of the gingiva was significantly thinner in ARP group (2.17 ± 0.54 mm) than in group SH (3.33 ± 0.99 mm; *p* < 0.05) (Figure 5a and Table 2).

#### 3.2.2. Tangential Thicknesses of the Buccal and Palatal Gingiva at 6 Months

The tangential thickness at the implant shoulder level (HT_0) on the buccal side was significantly thinner in group ARP (2.15 ± 0.47 mm) than in group SH (2.90 ± 0.76 mm; *p* < 0.05). Similar outcomes were observed at the palatal side (2.94 ± 0.74 mm vs. 3.79 ± 1.13; *p* < 0.05). The thickness at 2 mm below the implant shoulder (HT_2) on the buccal and palatal side was slightly thicker in ARP group (buccal: 1.53 ± 0.63 mm; palatal: 3.49 ± 0.86 mm) compared to group SH (buccal: 1.33 ± 0.41 mm; palatal: 3.26 ± 0.76 mm) without reaching statistical significance (*p* > 0.05) (Figure 5b and Table 2).

#### 3.2.3. Change of the Level of the MGJ on the Buccal Side from Suture Removal to 6 Months

The level of the buccal MGJ shifted in an opposite direction in the two groups (ARP and SH). The extent of change was minimal though (less than 1 mm). The MGJ moved apically in group ARP (0.63 ± 1.21 mm) whereas it shifted coronally in group SH (−0.29 ± 0.60 mm) (intergroup comparison *p* > 0.05) (Figure 5c).

## 4. Discussion

The present study evaluated the dimension of the soft tissues following tooth extraction in the posterior maxilla comparing ARP versus spontaneous healing at 6 months post-surgery. Main outcomes demonstrated: A significantly thinner crestal gingiva in group ARP group compared to group SH group; a significantly thinner tangential thickness of the buccal and palatal gingiva at the level of the virtually placed implant shoulder (HT_0) in group ARP compared to group SH. The thickness of the gingiva at the level 2 mm below the implant shoulder (HT_2) and the change of the MGJ on the buccal side did not differ significantly between the two groups.

Previous data suggested that the soft tissues increase in thickness following tooth extraction in the esthetic zone because of resorption of the underlying bone [10]. Based on a three-dimensional analysis, sites with a thin alveolar bone phenotype (thickness of 1 mm or less) had a greater tendency to resorption than sites with a buccal bone plate thicker than 1 mm [23]. In addition, the thickness of the labial gingiva tended to increase in thickness in cases with a thin bone biotype [11]. This phenomenon has in part been attributed to fibroblasts and myofibroblasts. Fibroblasts migrating to a wound area undergoing vertical bone resorption tend to differentiate to myofibroblast to stabilize the wound margin. This will eventually increase the gingival thickness at the extraction sites [10].

A similar biologic mechanism was likely observed in the present study, thereby resulting in an increase of the thickness of the crestal gingiva in group SH. In the previously published study, reporting on the dimensional changes of hard tissues, the crestal bone level at 6 months post-surgery was significantly higher (approximately 1.5-fold closer to coronal) in group ARP compared to group SH [6]. Gingival fibroblast might predominantly have occupied the area where crestal bone was lost in group SH, consequently increasing the gingival thickness. In contrast, the reduced thickness of the crestal gingiva observed in group ARP might be attributed to the relatively well-maintained crestal bone height.

The thickness of the peri-implant soft tissues has been reported to be 3 mm, a 1 mm increase compared to natural teeth, thereby fulfilling its function as a biologic barrier [15,24,25]. In case of an artificial decrease in dimension (experimental), spontaneous marginal bone resorption and recession occur to re-establish a sufficient height of the peri-implant mucosa [14]. Similar observations were also made clinically showing that the thicker the peri-implant mucosa, the less the marginal bone resorption [26]. Consequently, when performing ARP, 2 mm of crestal soft tissue thickness might be expected in the posterior zone. Even though ARP showed a significant benefit in terms of optimizing the vertical ridge dimension of bone [6], the present result in turn could be considered as a disadvantage in terms of supracrestal soft tissue thickness and would eventually result in a recommendation that dental implants should be placed with the implant shoulder 1 mm below the bone crest in order to guarantee a 3 mm vertical soft tissue thickness. In case of spontaneous healing, dental implants should be placed at the level of the bone crest, considering that 3 mm of vertical soft tissue thickness can be expected.

The tangential thickness of the buccal and palatal gingiva presented different patterns depending on the measured level (equivalent to or 2 mm below the implant shoulder). At the level of the implant shoulder, HT_0, the thickness of the gingiva was significantly thinner in group ARP compared to group SH, both, on the buccal as well as the palatal side. At the level, 2 mm below the virtually placed implant shoulder, the hard tissue dimension and the respective loss had a greater influence [6]. In the previous published data on changes of the hard tissue dimension, the horizontal ridge width on the buccal and palatal side at 6 months was comparable for both groups. As such, outcomes of the soft tissues were similar in both groups as well. When comparing HT_0 and HT_2 of the buccal side to those of the palatal side within each group, the palatal thickness of the gingiva was thicker than the one on the buccal side. This might be explained by the fact that the palatal soft tissues mainly consist of masticatory gingiva, usually having a thicker phenotype than the buccal gingiva [27].

Considering a mucosal thickness of 2 mm as a threshold for a thick peri-implant mucosal phenotype from an esthetic point of view [28], the observed thickness on the buccal and palatal side appear to be adequate. Similar to the vertical thickness of the mucosa on top of an implant, the tangential mucosal thickness covering the buccal and palatal bone are considered important in terms of maintaining the peri-implant health [29]. Since the palatal mucosa tends to have a thick phenotype in many cases, clinicians are usually concerned about the buccal mucosal thickness. A thicker buccal mucosa simplifies oral hygiene [30,31], which results in less peri-implant marginal bone loss and less esthetic complications (i.e., mucosal recession, mucosal discoloration) [29,32].

Both of the biomaterials grafted in ARP group contain xenogeneic collagen. Barrier membrane used in the present research was native, non-crosslinked, resorbable collagen membrane. Even though the present study did not include histologic evaluation, it could be easily speculated that the barrier membrane did not affect the thickness of the soft tissue, considering that non-crosslinked collagen degrades and gets thinner significantly 4 to 8 weeks after when it is applied intraorally, whereas crosslinked collagen membrane lasts longer [33,34]. Collagen added in the bone graft material also does not seem to have an influence on soft tissue remodeling. Previous pre-clinical animal experiment showed serial histologic images (1, 2, and 4 weeks) and revealed that collagen portion went through fast degradation from a week after grafting which consequently formed inter-particular space for enhancing new bone formation [35].

The level of the buccal MGJ migrated apically in group ARP and more coronally in group SH over 6 months. The suture performed to stabilize the blood clot in SH group might have pulled the buccal gingiva in the coronal direction and caused coronal migration of buccal MGJ, whereas this seemed not happened in the group ARP because the socket was filled with the graft materials and the wound was left open to induce secondary healing. The difference between the two groups, however, was minimal, not significant and not clinically relevant. Still, it implies ARP results in a clinical benefit in preserving the amount of keratinized tissue and simplifying implant placement by allowing for a flapless surgery. The prevention of a corono-apical migration of the buccal keratinized gingiva in case of a flapless ARP procedure was reported previously [18]. Although in that study, the hard tissue dimension was better preserved in cases of a flapped ARP procedure compared to a flapless surgery [18], a number of studies found that ARP with open healing showed similar results in terms of hard tissue dimensional changes compared to a flapped surgery [36,37,38]. This means that flapless ARP allows optimizing the outcomes on the hard and soft tissue level [6] in the posterior maxilla. Still, there is a controversy whether or not a certain amount of keratinized mucosa is beneficial in maintaining peri-implant health [32,39,40,41]. Patients, however, feel more comfortable performing adequate plaque control when a wider band of keratinized tissue is present around implants [42].

When the implants were vertically placed, total of 8 dehiscence defects were expected. In 7 out of 8 cases, the dehiscence was created on a palatal aspect (3 cases in ARP group; 4 cases in SH group). The reason why there were more defects expected in the palatal than the buccal could be explained according to the data of previous study [6]. It was reported that ARP did not show significant advantage in preserving the horizontal ridge dimension over SH, and at 6 months after the extraction, horizontal ridge width of the palatal side was slightly thinner than that of the buccal side, especially near the bone crest.

Interpretation of the current study data should be considered with care since the mucosal thickness was measured around virtually positioned implants. Although the position of the implants was planned in accordance with appropriate prosthesis and surrounding alveolar bone, this can be subjective, and the measurement outcomes may vary depending on the position of the implants. Moreover, it is the ultimate goal for clinicians to place the implant as planned, however, the surgical outcome may vary due to (i) any discrepancies between actual oral environment and CBCT image, (ii) surgical sensitivity, and/or (iii) physiological changes occur during the time CBCT was taken and the surgery. The authors believe that the measurements conducted with intra-oral scan and CBCT data collected at 6 months post-operative could eliminate human error that can occur during surgery or any physiological changes that can occur between the data collection and the surgery.

It might have been better if the change in the soft tissue dimension or volume comparison over time was also provided in this study. Even though the optical impression was taken two times (suture-removal and 6 months post-surgery), the STL achieved at suture-removal seemed inappropriate to use for the analysis based on the superimposition, since an indefinite crestal soft tissue profile was presented at the extracted site because of blood clots, which might have resulted in inaccurate results. This could be considered as a limitation of the present study, and it is recommended for the future study to obtain the STL data at the time points other than the suture-removal.

## 5. Conclusions

Within the limitation of this study, ARP performed in the posterior maxilla resulted in a thinner soft tissue dimension at the level of the virtually placed implant shoulder at 6 months after the extraction. The change of the level of the buccal MGJ was similar in both groups, ARP and spontaneous healing, for 6 months post tooth extraction.

## Figures and Tables

**Table 1:** Demographic information of the subjects evaluated.

Subjects Included for the Evaluation	Total 35	ARP SH	19 16
Age (years, mean ± standard deviation)	Total 53.2 ± 10.6	ARP SH	55.3 ± 8.33 50.8 ± 12.6
Gender	Male 24	ARP SH ARP SH	13 11 6 5
	Female 11		
Location of the site treated and evaluated	First molar 21	ARP SH ARP SH	14 7 5 9
	Second molar 14		
Dehiscence defects observed in virtual implant placement	Buccal 1	ARP SH ARP SH	0 1 (2 mm in height) 3 (1 mm in height) 4 (1 mm in height)
	Palatal 7		

**Table 2:** The measurements of gingival thickness (mm, mean ± standard deviation).

Group	Vertical Thickness of Crestal Gingiva	Tangential Thickness at the Implant Shoulder Level (HT_0)		Tangential Thickness at 2 mm Below the Implant Shoulder (HT_2)	
		Buccal	Palatal	Buccal	Palatal
ARP	2.17 ± 0.54 *	2.15 ± 0.47 *	2.94 ± 0.74 *	1.53 ± 0.63	3.49 ± 0.86
SH	3.33 ± 0.99 *	2.90 ± 0.76 *	3.79 ± 1.13 *	1.33 ± 0.41	3.26 ± 0.76

* (bold): statistical significance observed between ARP and SH groups (*p* < 0.05).
